# Acceptability of a Mobile Clinical Decision Tool Among Emergency Department Clinicians: Development and Evaluation of The Ottawa Rules App

**DOI:** 10.2196/10263

**Published:** 2018-06-11

**Authors:** Michelle Paradis, Ian Stiell, Katherine M Atkinson, Julien Guerinet, Yulric Sequeira, Laura Salter, Alan J Forster, Malia SQ Murphy, Kumanan Wilson

**Affiliations:** ^1^ Clinical Epidemiology Program Ottawa Hospital Research Institute Ottawa, ON Canada; ^2^ Department of Emergency Medicine University of Ottawa Ottawa, ON Canada; ^3^ Department of Public Health Sciences Karolinska Institute Karolinska Sweden

**Keywords:** emergency department medicine, clinical tools, mobile apps, digital health

## Abstract

**Background:**

The Ottawa Ankle Rules, Ottawa Knee Rule, and Canadian C-Spine Rule—together known as The Ottawa Rules—are a set of internationally validated clinical decision rules developed to decrease unnecessary diagnostic imaging in the emergency department. In this study, we sought to develop and evaluate the use of a mobile app version of The Ottawa Rules.

**Objective:**

The primary objective of this study was to determine acceptability of The Ottawa Rules app among emergency department clinicians. The secondary objective was to evaluate the effect of publicity efforts on uptake of The Ottawa Rules app.

**Methods:**

The Ottawa Rules app was developed and publicly released for free on iOS and Android operating systems in April 2016. Local and national news and academic media coverage coincided with app release. This study was conducted at a large tertiary trauma care center in Ottawa, Canada. The study was advertised through posters and electronically by email. Emergency department clinicians were approached in person to enroll via in-app consent for a 1-month study during which time they were encouraged to use the app when evaluating patients with suspected knee, foot, or neck injuries. A 23-question survey was administered at the end of the study period via email to determine self-reported frequency, perceived ease of use of the app, and participant Technology Readiness Index scores.

**Results:**

A total of 108 emergency department clinicians completed the study including 42 nurses, 33 residents, 20 attending physicians, and 13 medical students completing emergency department rotations. The median Technology Readiness Index for this group was 3.56, indicating a moderate degree of openness for technological adoption. The majority of survey respondents indicated favorable receptivity to the app including finding it helpful to applying the rules (73/108, 67.6%), that they would recommend the app to colleagues (81/108, 75.0%), and that they would continue using the app (73/108, 67.6%). Feedback from study participants highlighted a desire for access to more clinical decision rules and a higher degree of interactivity of the app. Between April 21, 2016, and June 1, 2017, The Ottawa Rules app was downloaded approximately 4000 times across 89 countries.

**Conclusions:**

We have found The Ottawa Rules app to be an effective means to disseminate the Ottawa Ankle Rules, Ottawa Knee Rule, and Canadian C-Spine Rule among all levels of emergency department clinicians. We have been successful in monitoring uptake and access of the rules in the app as a result of our publicity efforts. Mobile technology can be leveraged to improve the accessibility of clinical decision tools to health professionals.

## Introduction

Clinical decision rules attempt to reduce the uncertainty of medical decision making by standardizing the collection and interpretation of clinical data. These tools are derived from original research and incorporate 3 or more variables from clinical assessment or simple tests. These offer concrete yes/no answers and help clinicians with management decisions at the bedside [1-3].

The Ottawa Knee Rule [4], Ottawa Ankle Rules [5], and Canadian C-Spine Rule [6] are 3 internationally validated clinical decision rules that were developed to facilitate rapid detection of bone fractures upon entry into the emergency department (ED) and reduce unnecessary radiographic series. In various clinical trials, clinicians who applied the rules had a 28% reduction in ankle and 14% reduction in foot radiographic series [7]. In addition to reducing unnecessary use of diagnostic imaging services, appropriate application of the rules has been shown to improve standardization of practice and care and reduce emergency room wait times with significant health cost savings [8-10]. Although clinical decision rules such as The Ottawa Rules were developed to assist with bedside diagnostic or therapeutic decisions, some may have limited impact due to weak clinician uptake in jurisdictions [11,12].

Use of mobile technologies in the health care setting has provided clinicians with a means of rapidly and easily accessing hospital information systems and services. Mobile apps now assist clinicians with day-to-day tasks including health record maintenance, patient management and monitoring, and medical education and training. There exists an opportunity to leverage increasing use of mobile devices to support easy and efficient access to clinical decision rules.

In this study, we sought to develop and evaluate a mobile app housing 3 validated ED clinical decision rules, collectively known as The Ottawa Rules. Our primary objective was to determine acceptability of a mobile app format of The Ottawa Rules among ED clinicians (physicians, residents, nurses, and medical students) at The Ottawa Hospital (TOH), in Ottawa, Canada. The secondary objective was to evaluate the effect of publicity efforts on uptake of The Ottawa Rules app.

## Methods

### Mobile App Development

The Ottawa Rules app was developed natively for both iOS and Android operating systems and as a mobile-enhanced website at www.theottawarules.ca. App development was led by The Ottawa Hospital mHealth Lab located at the Ottawa Hospital Research Institute (OHRI). The Ottawa Knee Rule, Ottawa Ankle Rules, and Canadian C-Spine Rule, together known as The Ottawa Rules, were included in the app. Each rule was made available as a set of images with clear procedural guidelines. Instructional videos and links to academic resources for each rule were also included. The app was designed to include a mechanism for feedback and support where users were permitted to provide suggestions for app improvements, report bugs, and request technical assistance.

### Pilot-Testing

Following internal alpha-testing, the prototype was beta-tested on 6 consenting ED clinicians at TOH. Beta testers were instructed to use the app as if they were experiencing the circumstances outlined in provided mock clinical scenarios. Scenarios involved a patient entering the ED with a suspected knee, foot, or neck injury for which the clinicians needed to use the app to determine whether the injury would require diagnostic imaging. Beta testers completed their mock scenario twice; once as new users and once after having familiarized themselves with the app for approximately 10 minutes. Semistructured interviews with beta testers were then conducted to establish further insight into what content would be appropriate for the app and any barriers to app access or perceived barriers for app use. Findings from beta-testing were then integrated into the app before its public release.

### App Release

The app was publicly released on April 21, 2016, for iOS devices via the App Store, for Android devices via Google Play, through OHRI’s app portal, and on the Web at www.theottawarules.ca. A press release was circulated on the same day with local and national news sources, national and international emergency medicine media, and through social media messaging on affiliate Twitter accounts. Social media posts about app release were also disseminated via affiliate Twitter accounts and Facebook pages, and announcements were made at departmental rounds.

### Study Enrollment

The study was advertised through poster displays in EDs at TOH Civic and General campuses and email distribution to ED staff and medical students at the University of Ottawa. Study staff also showcased the new app at a hospital-organized digital health networking event, resident rounds, and the hospital’s annual academic research day. Primary study recruitment was conducted in person by study staff. Participants were required to be over 18 years of age, work in or be on rotation in the ED, possess an institutional email (TOH, OHRI, or University of Ottawa), and own a personal or institutional iOS or Android mobile phone onto which they could download The Ottawa Rules app. Since 2011, TOH has been equipping clinicians with iPads—clinicians also had the option of downloading The Ottawa Rules app onto these institutional devices and enrolling in the study.

**Figure 1:**
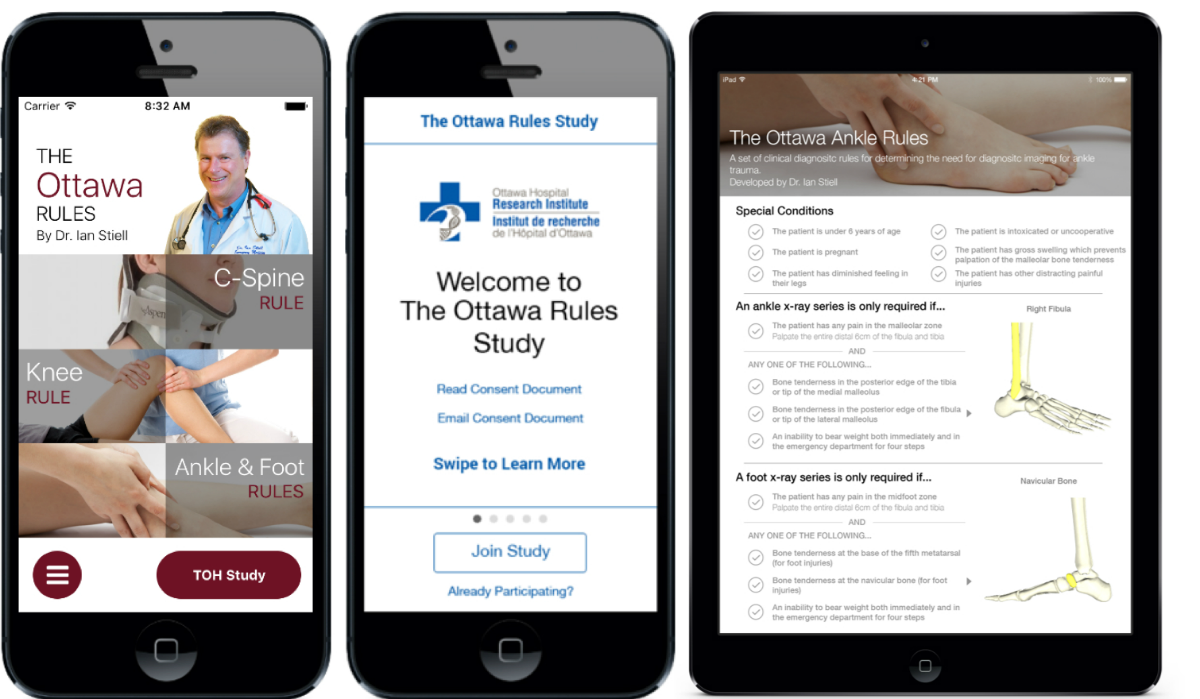
The Ottawa Rules interfaces on Android and iOS.

Inclusion criteria were assessed as part of the in-app informed consent process. Figure 1 provides screen shots of The Ottawa Rules app home screen with the “TOH Study” button for participants, informed consent screen, and 1 of the 3 in-app guidelines. From the app’s home screen interface, users could select 1 of the 3 rules according to the patient’s suspected injury. From the main menu, there were options to learn about The Ottawa Rules app, link to the lead physician’s Twitter account, provide feedback, and read the terms of use. With the goal of enhancing app usefulness, study participants had additional access to hospital resources within the app; these features were not available to general app users. Hospital resources included TOH’s antibiotic guidelines, nursing medical directives, and triage algorithms.

Once enrolled, consenting participants had to verify their institutional email before proceeding. Participants had in-app access to the consent documents and contact information of study staff throughout the duration of the study. Participants were instructed to explore the app features and use the app as reasonable when evaluating patients with suspected knee, foot, or neck injuries. This study was approved by the Ottawa Health Research Network Science Research Ethics Board (#20150405-01H).

### Data Collection

Frequency of use of The Ottawa Rules app was measured via in-app analytics and user surveys as detailed below.

#### Participant Survey

One-month post-study enrollment, participants were prompted via their verified institutional email to complete a usability survey to assess their perceived acceptability and usability of the app. The survey consisted of multiple-choice, 5-point Likert scale, and open-ended questions designed to ascertain participant demographics, ease of use, and intention for future use and provide the opportunity for written feedback. Participants who completed the end-study survey received an electronic coffee gift card in the amount of Can $10 (US $8).

#### Technology Readiness Index

The Technology Readiness Index (TRI) 2.0 was also administered in the 1-month survey. The TRI 2.0 includes 16 questions that measure an individual’s innate “propensity to embrace and use new technologies for accomplishing goals in home, life, and at work.” TRI questions are measured on a 5-point Likert scale ranging from 1=strongly disagree to 5=strongly agree and capture 4 dimensions. Two motivating dimensions capture qualities of individual optimism and innovativeness, whereas questions pertaining to individual discomfort and insecurity are considered inhibiting dimensions. A higher individual TRI indicates a higher likelihood to adopt new technology: TRI 2.0 = (innovativeness + optimism + [6 – discomfort] + [6 – insecurity]) / 4). Questions related to each of the 4 TRI 2.0 dimensions are provided in Multimedia Appendix 1.

#### Participant In-App Activity

Analytics on participant use of the app were encrypted and sent to a secure cloud server in Canada administrated by The Ottawa Hospital mHealth Lab at OHRI. The following metrics were collected on an individual level, only identified by study ID on the server: date app was first opened, number of times each rule was accessed, number of consenting participants who did not reopen the app, frequency of rule use, number of app sessions, and number and content of submitted feedback reports.

#### Google Analytic Data

General anonymous usage data of The Ottawa Rules app were collected through Google Analytics and used to gauge global app uptake and success of promotional activities. Google Analytics was used to ascertain total downloads, geographical region by IP address, app screens accessed, and average time spent in the app.

## Results

### Study Participants

**Figure 2:**
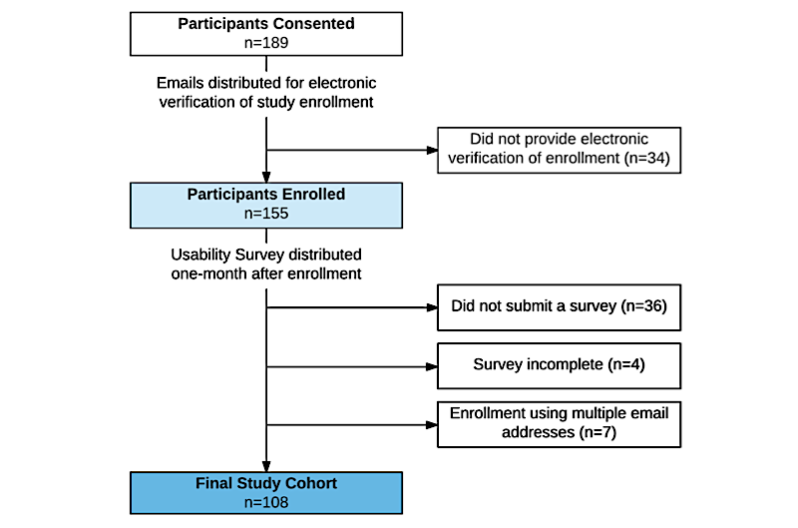
Study flow diagram.

A total of 155 consenting participants provided electronic verification of their enrollment into the study, and 119 (76.8%) participants submitted usability surveys 1 month after study enrollment. Due to data quality concerns, participants who enrolled under multiple email addresses and those who did not complete the usability survey in full were excluded from analysis. The final study cohort consisted of 108 participants (Figure 2).

Nurses constituted the largest number of study participants (42/108, 38.9%), followed by residents (33/108, 30.6%), physicians (20/108, 18.5%), and medical students (13/108, 12.0%). The majority of participants were 34 years of age or younger (73/108, 68.2%) and owned iOS devices (87/108, 80.6%). Table 1 summarizes the baseline characteristics of study participants.

### Usability Survey

**Figure 3:**
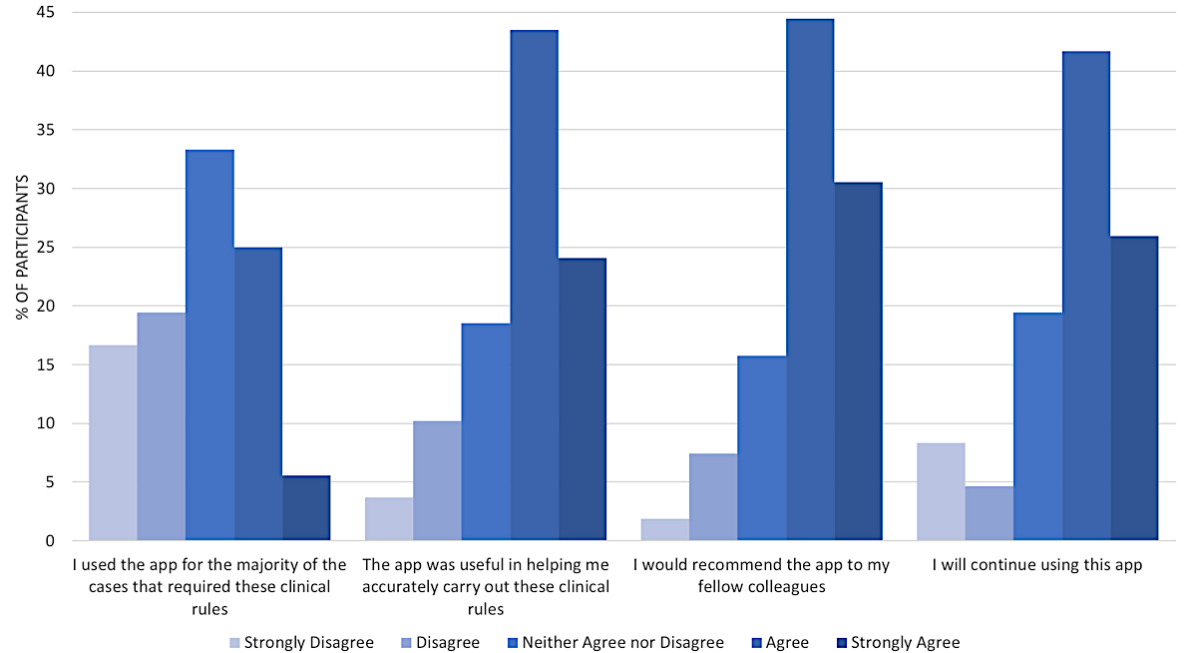
Usability survey responses.

When asked if they encountered any issues using the app, all participants reported either strongly disagree (103/108, 95.4%) or disagree (5/108, 4.6%). Self-reported frequency of use revealed the majority of participants (92/108, 85.2%) used the app at least once during the course of the study. A total of 43.5% (43/108) of participants said they used the app weekly, 38.9% (42/108) said monthly, 14.8% (16/108) said never, and 2.8% (3/108) said they used the app daily (Figure 3).

Questions on app usability revealed favorable reception by ED clinicians. A total of 30.6% (33/108) of participants indicated strongly agree or agree when asked if they used the app for the majority of the cases that required application of the clinical decision rules, 67.6% (73/108) of participants found the app useful in applying the clinical rules (strongly agree, agree), 75.0% (81/108) indicated that they would recommend the app to colleagues, and 67.6% (73/108) would continue using the app in its current form. Figure 3 summarizes usability survey data.

In addition, 56.5% (61/108) of participants provided free-form written feedback. Feedback included recommendations based on functionality, acceptability, and available features. Participants reported a desire for access to more TOH-developed decision aids, particularly the addition of the Canadian CT Head Rule and Subarachnoid Hemorrhage Risk Score. Users also indicated wanting access to more institutional resources and directives. A number of participants suggested inclusion of more interactive features, including a drop-down menu for easier and quicker navigation. Some indicated their preference for other medical mobile apps that housed more comprehensive or interactive lists of medical directives and decision rules. Lastly, participants highlighted the potential benefits of the use of the app among early learners or community physicians who might be less familiar with The Ottawa Rules.

**Table 1:** Summary of participant characteristics (n=108).

Characteristic		Value	
Age^a^ (years), mean (SD)		33.7 (9.9)	
**Age range (years), n (%)**			
	18-24		9 (8.4)
	25-34		64 (59.8)
	35-44		14 (13.1)
	45-54		14 (13.1)
	55-64		6 (5.6)
**Sex, n (%)**			
	Male		49 (45.4)
	Female		59 (54.6)
**Level of training, n (%)**			
	Medical student		13 (12.0)
	Nurse		42 (38.9)
	Resident		33 (30.6)
	Physician		20 (18.5)
Years of service, mean (SD)		6.85 (7.8)	
**Years of service, n (%)**			
	0-4		61 (56.4)
	5-9		22 (20.4)
	10-14		7 (6.5)
	15-19		7 (6.5)
	20-24		4 (3.7)
	>25		7 (6.5)
**Mobile operating system used, n (%)**			
	iOS		87 (80.6)
	Android		21 (19.4)

^a^One participant did not report a valid age; this person’s age data is not reported.

### Technology Readiness

After the exclusion of 6 incomplete surveys, the median TRI score was 3.56 (interquartile range [IQR] 0.62) out of a maximum score of 5, suggesting a slightly higher than average (3.02) propensity for technological adoption among participants. Examination of TRI scores across ED staff role (nurse, physician, resident, medical student), age (<35 or ≥35 years), by self-reported frequency of app use, and by response to usability questions revealed no association between TRI scores and patient demographics or user satisfaction and use of The Ottawa Rules app. The distribution of participant responses to survey questions ascertaining propensity to adopt new technologies overall and by participant subgroup are provided in Multimedia Appendix 1.

### In-App Activity

Study participants accessed the app a total of 762 times between April 21 and August 30, 2016 (Table 2). Server data showed that of the 108 participants, 13 (12.0%) participants did not venture beyond the app home screen to use specific app features, confirming patterns of self-reported use. Nurses were the most active users of the app features (responsible for 37.8% [288/762] of activity), followed closely by ED residents (32.5% [248/762] of activity). Of the 3 Ottawa Rules hosted in the app, the Canadian C-Spine Rule was the most frequently accessed overall (20.1% [153/762] of app events), followed by the Ottawa Ankle Rules (134/762, 17.6%), and Ottawa Knee Rule (128/762, 16.8%). TOH guidelines and algorithms accounted for 45.5% (347/762) of app events overall and were among the most accessed tools across all participant subgroups.

**Table 2:** In-app activity of study participants overall and by clinician role.

	Overall, n (%)	Students, n (%)	Residents, n (%)	Nurses, n (%)	Physicians, n (%)
Total number of app events	762 (100)	121 (15.9)	248 (32.5)	288 (37.8)	105 (13.8)
Ankle Rules events	134 (17.6)	21 (17.4)	46 (18.6)	49 (17.0)	18 (17.1)
Knee Rule events	128 (16.8)	22 (18.2)	37 (14.9)	48 (16.7)	21 (20.0)
C-Spine Rule events	153 (20.1)	26 (21.5)	46 (18.6)	47 (16.3)	34 (32.4)
Other TOH^a^ resources	347 (45.5)	52 (43.9)	119 (47.9)	144 (50.0)	32 (30.5)

^a^TOH: The Ottawa Hospital.

**Table 3:** App activity from April 21, 2016, to June 1, 2017.

Metric		Amount, n (%)	
**Total downloads (n=3863)**			
	iOS		1853 (47.9)
	Android		1638 (42.4)
	Other		372 (9.7)
**Downloads by country (n=3863)**			
	Canada		1234 (31.9)
	United States		953 (24.7)
	Spain		347 (8.9)
	Other		1329 (34.5)
**In-app events (n=7747)**			
	Ottawa Ankle Rules		2066 (26.7)
	Ottawa Knee Rule		1991 (25.7)
	Canadian C-Spine Rule		1904 (24.6)
	Additional features^a^		1786 (23.0)

^a^Additional features includes other screens accessible from the app homepage or main menu (eg, Terms of Use, About).

A summary of global app use and in-app activity since public release is shown in Table 3. As of June 1, 2017, The Ottawa Rules app had been downloaded nearly 4000 times across 89 countries. Users in Canada accounted for the largest number of downloads (1234/3863, 31.9%), followed by users in the United States (953/3863, 24.7%) and Spain (347/3863, 8.9%). The Ottawa Ankle Rules were accessed 2066 times, followed by the Ottawa Knee Rule (1991 times) and Canadian C-Spine Rule (1904 times).

Uptake of the app across 89 countries worldwide may reflect success of promotional efforts of our team for The Ottawa Rules app. Such efforts included media releases [13], a local morning televised news broadcast, and dissemination of the work through academic and clinical channels. We observed an increase in app downloads following circulation of a press release on May 9, resulting in 202 daily downloads. The period between September 23-30, 2016, saw 171 new downloads in Spain. Other potential reasons for global success may be app usefulness spread through word of mouth between clinicians and interactions or presentations at national and international conferences.

## Discussion

### Principal Findings

Mobile apps can be leveraged to improve the accessibility of clinical decision rules in the ED. Pilot-testing of The Ottawa Rules app among ED workers produced useful feedback that can be used to optimize the platform for our users. Overall, survey data suggest the app was useful in guiding clinical decision making and is a tool that clinicians would use in the future and recommend to others. A preference for single-source access to clinical resources and inclusion of additional decision rules and center-specific directives were among the most frequently cited feedback responses. Users also indicated that the platform could be improved by inclusion of interactive features.

The Ottawa Ankle Rules, Ottawa Knee Rule, and Canadian C-Spine Rule were developed to reduce unnecessary radiography for ankle/foot, knee, and cervical spine injuries without jeopardizing patient care. The rules have been widely validated across numerous international patient and hospital settings [14-17] and have been shown to reduce department crowding and patient length of stay [18] and save on hospital resources [19]. Dissemination, uptake, and implementation of the rules have not been optimal, however, even with educational strategies [12,20].

Health care has been impacted by advances in mobile technology. Whereas previously pagers and personal digital assistants were commonplace, mobile phones and tablets are now the preferred computing devices for health care professionals [21]. Ownership and use of smart devices is particularly high among the health care community, who report using medical apps on personal devices for both clinical activities and continued education [22,23]. Health care professionals increasingly rely on electronic resources to support patient management decisions, and there are numerous apps available that help provide information on diagnosis, treatment, and standard clinical formulas [24]. Other mobile apps, similar to the one evaluated in this study, have been designed to help clinicians identify the most appropriate scans or tests to order, thereby increasing efficiency of use of hospital resources [24]. Medical apps, although convenient, must be viewed with caution, however. Despite the high rate of adoption of health apps among young health care providers, medical professional involvement in the development of such apps is notably limited [23]. As a result there is a paucity of literature for reference on the rate of adoption, satisfaction, and use scores for apps used by health care providers. With the number of such tools becoming publicly available, the source of information upon which medical apps and electronic resources have been developed must be considered. Standards for the evaluation and validation of medical apps are warranted to ensure that the recommendations and outputs from these tools are reliable and safe [25].

### Strengths and Limitations

This study has important strengths and weaknesses. Strengths include close involvement of clinicians in the development of The Ottawa Rules app, user testing among all levels of ED staff, the use of surveys to capture both closed and open-ended feedback on the app system, and a high response rate (119/155, 76.8%). The successful completion of this study with over 100 ED workers of varied educational training, duration of employment, and age demonstrated an openness of the clinical community to trying new technology in the workplace. Our ability to leverage 3 separate data collection mechanisms to capture user-level (study participants) and aggregate-level (all app users) app activity is also a notable strength. Self-reported use of The Ottawa Rules app by participants, validated by server analytics data, confirmed that 12% to 15% of participants did not actively use the app despite having it downloaded on their phones. Through server analytics, we were able to identify the most frequently accessed app features and resources. Google Analytics data further permitted review of in-app activity among all users of the app, beyond the study cohort. Through Google Analytics, patterns of download across geographies and by type of devices were available for review, and trends in app activity following promotional efforts could be assessed. The primary weaknesses of this study were the small sample size, self-selecting nature of participant recruitment, and our single-center approach. This study also took place at a tertiary care hospital in an urban center where there was a high degree of familiarity with The Ottawa Rules among ED workers. As highlighted by some participants, evaluation of receptiveness to the app among medical trainees, general practitioners, and community physicians may be warranted, as these groups are less likely to be familiar with the rules.

### Conclusion

As we seek to optimize The Ottawa Rules app based on feedback to improve the user experience, interactive modalities will be provided. Importantly, inclusion of new clinical decision rules and other resources will be incorporated to provide a more comprehensive tool to our users. Work in this field would benefit from ongoing clinician involvement in the development and evaluation of health apps to ensure app quality, reliability, and user satisfaction. Future work should aim to assess the impact of health apps such as The Ottawa Rules on the quality and cost effectiveness of patient care and hospital resources. In sum, The Ottawa Rules app stands to provide health care professionals an efficient, reliable, and user-friendly means of accessing clinically validated decision rules shown to reduce health care costs and improve quality of patient care.

## Appendix

Multimedia Appendix 1 Survey questions and response data.

## Abbreviations

ED: emergency department
IQR: interquartile range
OHRI: Ottawa Hospital Research Institute
TOH: The Ottawa Hospital
TRI: Technology Readiness Index
