# Supplementary material for: Acceptability of a Mobile Clinical Decision Tool Among Emergency Department Clinicians: Development and Evaluation of The Ottawa Rules App
Source: JMIR Mhealth Uhealth. 2018 Jun 11;6(6):e10263. doi: 10.2196/10263 (PMC6018230; doi:10.2196/10263)
Supplement: Multimedia Appendix 1 [file mhealth_v6i6e10263_app1.pdf]

Supplementary Materials

Table 1. Survey questions used to ascertain participant TRI

**Optimism**	**Innovativeness**
New technologies contribute to a better quality of life (OPT1)	Other people come to me for advice on new technologies (INN1)
Technology gives me more freedom of mobility (OPT2)	In general, I am among the first in my circle of friends to acquire new technology when it appears (INN2)
Technology gives people more control over their daily lives (OPT3)	I can usually figure out new high-tech products and services without help from others (INN3)
Technology makes me more productive in my personal life (OPT4)	I keep up with the latest technological developments in my areas of interest (INN4)
**Discomfort**	**Insecurity**
When I get technical support from a provider of a high-tech product or service, I sometimes feel as if I am being taken advantage of by someone who knows more than I do (DIS1)	People are too dependent on technology to do things for them (INS1)
Technological support lines are not helpful because they don't explain things in terms I understand (DIS2)	Too much technology distracts people to a point that is harmful (INS2)
Sometimes, I think that technology systems are not designed for use by ordinary people (DIS3)	Technology lowers the quality of relationships by reducing personal interaction (INS3)
There is no such thing as a manual for a high-tech product or service that's written in plain language (DIS4)	I do not feel confident doing business with a place that can only be reached online (INS4)

Table 2. Results of technology readiness survey

	**Optimisim**	**Innovativeness**	**Discomfort**	**Insecurity**	**TRI**
Mean (SD)	4.08 (0.72)	3.42 (0.79)	2.29 (0.73)	2.97 (0.83)	3.56 (0.54)
Median (IQR)	4 (3.75, 4.75)	3.38 (2.75, 4.00)	2.25 (1.75, 3.00)	3.00 (2.31, 3.50)	3.56 (3.19, 3.88)

Table 3. TRI by participant subgroups

**Participant role in the ED**	**Mean TRI (SD)**	**P-Value**
Nurse (n=41)	2.78 (0.42)	0.47
Physician (n=18)	2.65 (0.49)	
Resident (n=31)	2.69 (0.43)	
Student (n=12)	2.85 (0.23)	
Participant Age		
<35 (n=70)	2.73 (0.39)	0.88
≥35 (n=31)	2.77 (0.50)	
Self-reported frequency of app use		
Never (n=16)	2.58 (0.51)	0.46
Daily (n=2)	2.63 (0.18)	
Weekly (n=43)	2.82 (0.42)	
Monthly (n=41)	2.72 (0.38)	
I used the app for the majority of the cases that required these clinical rules		
Strongly Disagree	2.58 (0.51)	0.07
Disagree	2.82 (0.40)	
Neutral	2.71 (0.35)	
Agree	2.83 (0.45)	
Strongly Agree	2.65 (0.42)	
The app was useful in helping me accurately carry out these clinical rules		
Strongly Disagree	2.38 (0.14)	0.90
Disagree	2.48 (0.48)	
Neutral	2.84 (0.49)	
Agree	2.78 (0.33)	
Strongly Agree	2.75 (0.47)	
I would recommend the app to my fellow colleagues		
Strongly Disagree	2.50 (0.35)	0.84
Disagree	2.59 (0.72)	
Neutral	2.75 (0.22)	
Agree	2.76 (0.37)	
Strongly Agree	2.75 (0.47)	
I will continue using this app		
Strongly Disagree	2.5 (0.64)	0.41
Disagree	2.69 (0.24)	
Neutral	2.74 (0.40)	
Agree	2.75 (0.36)	
Strongly Agree	2.81 (0.46)	

Data compared by Kruskal-wallis test.
